# Kleeb Bua Daeng, a Thai Traditional Herbal Formula, Ameliorated Unpredictable Chronic Mild Stress-Induced Cognitive Impairment in ICR Mice

**DOI:** 10.3390/molecules24244587

**Published:** 2019-12-14

**Authors:** Juthamart Maneenet, Supawadee Daodee, Orawan Monthakantirat, Chantana Boonyarat, Charinya Khamphukdee, Pakakrong Kwankhao, Supaporn Pitiporn, Suresh Awale, Yaowared Chulikhit, Anake Kijjoa

**Affiliations:** 1 Graduate School of Pharmaceutical Sciences, Khon Kaen University, Khon Kaen 40002, Thailand; juthamart_pp@hotmail.com; 2 Division of Pharmaceutical Chemistry, Faculty of Pharmaceutical Sciences, Khon Kaen University, Khon Kaen 40002, Thailand; csupawad@kku.ac.th (S.D.); oramon@kku.ac.th (O.M.); chaboo@kku.ac.th (C.B.); charkh@kku.ac.th (C.K.); 3 Department of Pharmacy, Chao Phya Abhaibhubejhr Hospital, Ministry of Public Health, Prachinburi, Thailand 25000, Thailand; pakakrong2@gmail.com (P.K.); spitiporn@yahoo.com (S.P.); 4 Division of Natural Drug Discovery, Institute of Natural Medicine, University of Toyama, 2630 Sugitani, Toyama 930-0194, Japan; suresh@inm.u-toyama.ac.jp; 5 *ICBAS*-Instituto de Ciências Biomédicas Abel Salazar and CIIMAR, Rua de Jorge Viterbo Ferreira, 228, 4050-313 Porto, Portugal

**Keywords:** herbal formula, unpredictable chronic mild stress, learning and memory behaviors, oxidative stress, *Piper nigrum*, *Centella asiatica*, *Nelumbo nucifera*

## Abstract

Thai traditional herbal formula ‘’Kleeb Bua Daeng (KBD)’’consists of a 1:1:1 ratio (dry weight) of three medicinal plants: *Piper nigrum* fruit, the aerial part of *Centella asiatica* and the petals of *Nelumbo nucifera*. Oral administration of KBD to unpredictable chronic mild stress (UCMS) mice significantly improved their cognitive function caused by chronic mild stress. Daily administration of KBD significantly decreased the serum corticosterone (CORT) and malondialdehyde (MDA) levels but increased the catalase and superoxide dismutase activities in both frontal cortex and hippocampus. The effects of KBD were similar to those caused by oral administration of vitamin E. HPLC analysis of the KBD extract revealed the presence of piperine, madecassoside, asiaticoside, luteolin-7-*O*-glucoside, rutin, kaempferol-3-glucoside, quercetin, kaempferol and ferulic acid as major constituents.

## 1. Introduction

Accumulated evidences demonstrated that chronic stress negatively affects neural plasticity and neurogenesis which produce depressive symptom, impairment in memory and learning processes [1,2]. The deleterious effect of chronic stress is modulated by the stress hormone, cortisol/corticosterone (CORT), neurotrophins, and various neurotransmitters [3,4,5]. The repeated stress also induces the overproduction of free radicals and the suppression of antioxidant system, leading to the brain oxidative damage [6,7]. The hippocampus is commonly implicated in stress-triggered hippocampal atrophy, and impaired synaptic plasticity [8]. Based on these reports, the unpredictable chronic mild stress (UCMS) animal model has been developed to mimic pathophysiological changes relevant to depression and memory decline. To obtain this effect, mice are chronically exposed to the micro-stressors for 5–9 weeks [9,10].

Kleeb Bua Daeng (KBD) is a multi-herbal formula used in the traditional Thai medicine for more than 70 years by local healers for the treatment of insomnia and memory impairment. KBD consists of three medicinal plants, i.e., *Piper nigrum* fruit, the aerial part of *Centella asiatica* and the petals of *Nelumbo nucifera*, in a 1:1:1 ratio (dry weight). Since June 2013, KBD, in the form of capsules, has been officially prescribed to promote brain health and relieve anxiety at Chao Phya Abhaibhubejr Hospital, where the traditional medicine is integrated in the modern therapy. The therapeutic effects of KBD could be justified by the medicinal properties of its plant ingredients. The methanolic extract of *P. nigrum* fruits was reported to enhance the cognitive function in the β-amyloid (1-42)-induced spatial memory impairment rat model by increasing the catalase, superoxide dismutase, glutathione peroxidase activities, as well as to reduce the level of malondialdehyde (MDA) [11]. On the other hand, *C. asiatica* was described to have cognitive enhancing properties by improving the antioxidant status, enhancing the nuclear factor erythroid 2-related factor 2 (Nrf2)-antioxidant response pathway, decreasing the oxidative stress and inhibiting the proinflammatory enzyme, phospholipase A2 [12,13]. *N. nucifera* was found to exhibit antidepressant, anti-anxiety, and cognitive enhancement activities. Moreover, the extracts of its rhizome and embryo seeds also showed significant improvements in memory function and neurogenesis by increasing cell proliferation in the rat dentate gyrus and inhibition of acetylcholinesterase (AChE) activity [14,15,16]. 

Because of the multi-pathogenesis of UCMS-induced cognitive impairment, the classical approach that modulates only one target may be insufficient. Thus, KBD, a combination of these three medicinal plants, could provide more additional benefits for dementia therapy. Since there is still no scientific-based evidences on the neuropharmacological effects of KBD, the objective of this study is to elucidate the effect of KBD on UCMS-induced cognitive dysfunction as well as to demonstrate a plausible mechanism underlying these effects. In order to warrant the results obtained from the neuropharmacological studies, HPLC fingerprints of the KBD extract and the extracts of each of the ingredient plants were also performed to identify the main compounds present in the KBD extract.

## 2. Results

### 2.1. Effect of KBD on UCMS-Induced Cognitive Deficits

**Figure 1:**
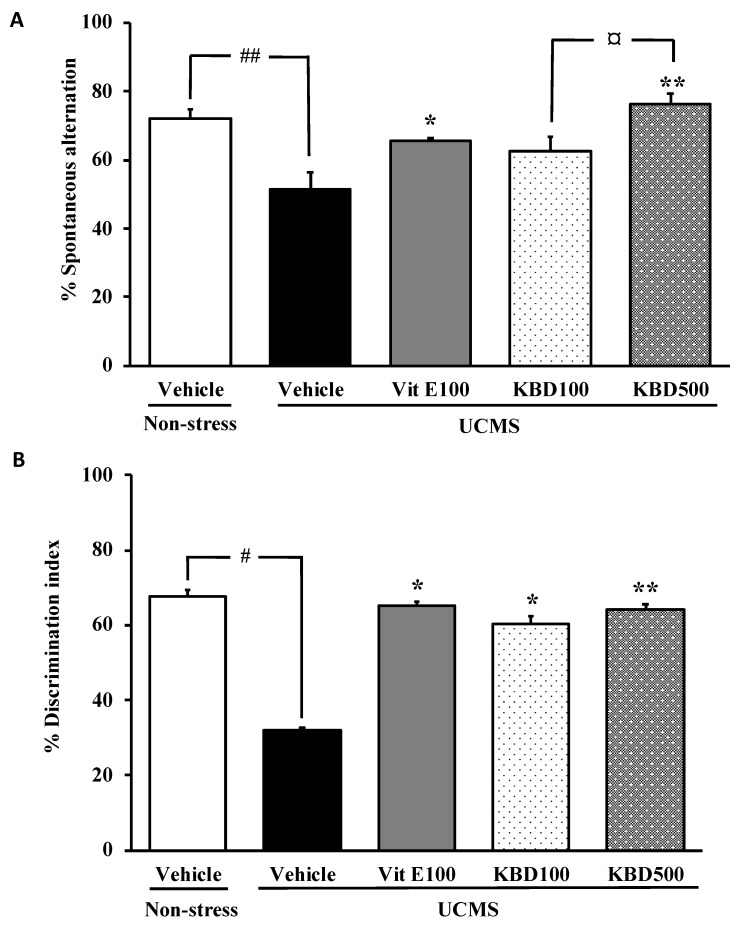
The effect of Kleeb Bua Daeng (KBD) and vitamin E on the unpredictable chronic mild stress (UCMS)-induced cognitive impairment in the Y-maze test (**A**) and the novel object recognition test (NORT) (**B**). Each column represents the mean ± S.E.M. (n = 8–10). ^#^ *p* < 0.05, ^##^ *p* < 0.001 vs. the vehicle-treated non-stress group. * *p <* 0.05, ** *p* < 0.001 vs. the vehicle-treated UCMS group. ^¤^ *p* < 0.05 compared between doses of KBD.

To determine whether KBD modulates the cognitive function in the unpredictable chronic mild stress (UCMS) model, spatial and non-spatial working memory performances of UCMS mice were performed in the Y-maze test and the novel object recognition test (NORT), respectively. The vehicle-treated UCMS mice exhibited significantly less percentage of spontaneous alternation than the non-stress group, indicating impairment of spatial working memory caused by UCMS. The KBD-treated UCMS groups showed significantly improved spontaneous alternation performance when compared with the vehicle-treated UCMS mice in a dose-dependent manner, which is similar to that treated with vitamin E (Figure 1A). The NORT also revealed the UCMS-induced cognitive dysfunction in mice. In the test trials, the non-stress group spent significantly more time exploring the novel object than the familiar object, whereas the UCMS mice failed to discriminate between the novel and familiar objects, indicating the UCMS-induced non-spatial working memory dysfunction. The UCMS mice that received vitamin E (100 mg/kg/day) and KBD (100 and 500 mg/kg/day) for 3 weeks showed significantly improved discrimination performance when compared to the UCMS-treated mice (Figure 1B).

### 2.2. Effect of KBD on the UCMS-Induced Hypersecretion of the Serum Corticosterone (CORT)

**Figure 2:**
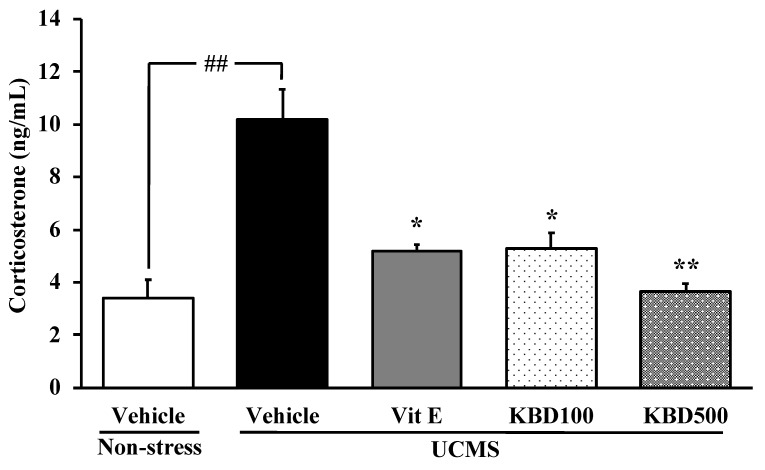
Effects of KBD and vitamin E on the UCMS-induced elevation in serum corticosterone (CORT) level. Each column represents the mean ± S.E.M (n = 3). ^##^ *p* < 0.001 vs. the vehicle-treated non-stress group. ^*^ *p* < 0.05, ^**^ *p* < 0.001 vs. the vehicle-treated UCMS group.

The level of the serum CORT was determined to clarify whether the feedback mechanism in the HPA axis was impaired by the UCMS exposure. The vehicle-treated UCMS group showed a significant increase in the serum CORT when compared to the vehicle-treated non-stress group. However, the UCMS-induced enhancement of serum CORT level was significantly suppressed by daily administration of KBD at doses of 100 and 500 mg/kg/day, as well as by vitamin E at a dose of 100 mg/kg/day (Figure 2).

### 2.3. Effect of KBD on the UCMS-Induced Lipid Peroxidation in the Brain

**Figure 3:**
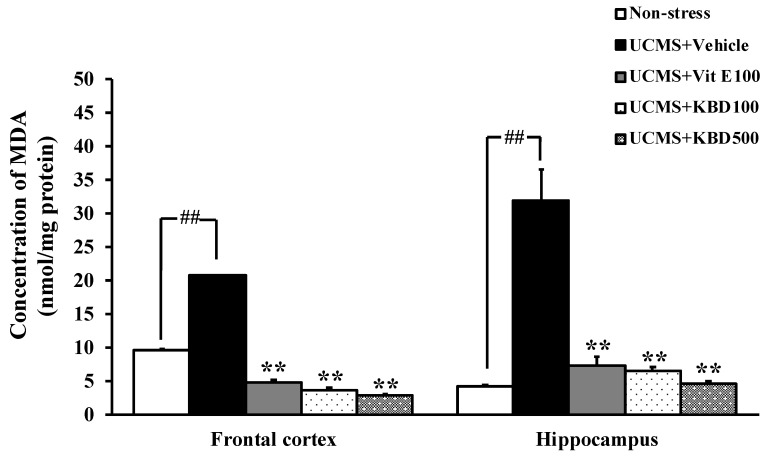
Effect of KBD and vitamin E on the UCMS-induced lipid peroxidation in mice frontal cortex and hippocampus. The lipid peroxidation in brain homogenate was determined using malondialdehyde (MDA) as a standard, and was expressed as nmol MDA/mg protein. Each data column represents the mean ± S.E.M. (n = 3–5). ^##^ *p* < 0.001 vs. the vehicle-treated non-stress group. ^**^ *p* < 0.001 vs. the vehicle-treated UCMS group.

In order to investigate the possible involvement of the brain oxidative stress in the UCMS-induced learning and memory impairments, as well as the effect of KBD on these symptoms, the levels of MDA, a biomarker for the lipid peroxidation, in the frontal cortex and hippocampus were examined. The UCMS was found to induce a significant increase in the MDA levels in both brain regions when compared with those in the non-stress control mice. Repeated administration of KBD at the doses of 100 and 500 mg/kg or vitamin E, at the dose 100 mg/kg/day, for 3 weeks significantly reduced the MDA levels in the frontal cortex and hippocampus of the UCMS mice (Figure 3).

### 2.4. Effect of KBD on UCMS-Reduced Brain Superoxide Dismutase (SOD) and Catalase (CAT) Activities 

**Figure 4:**
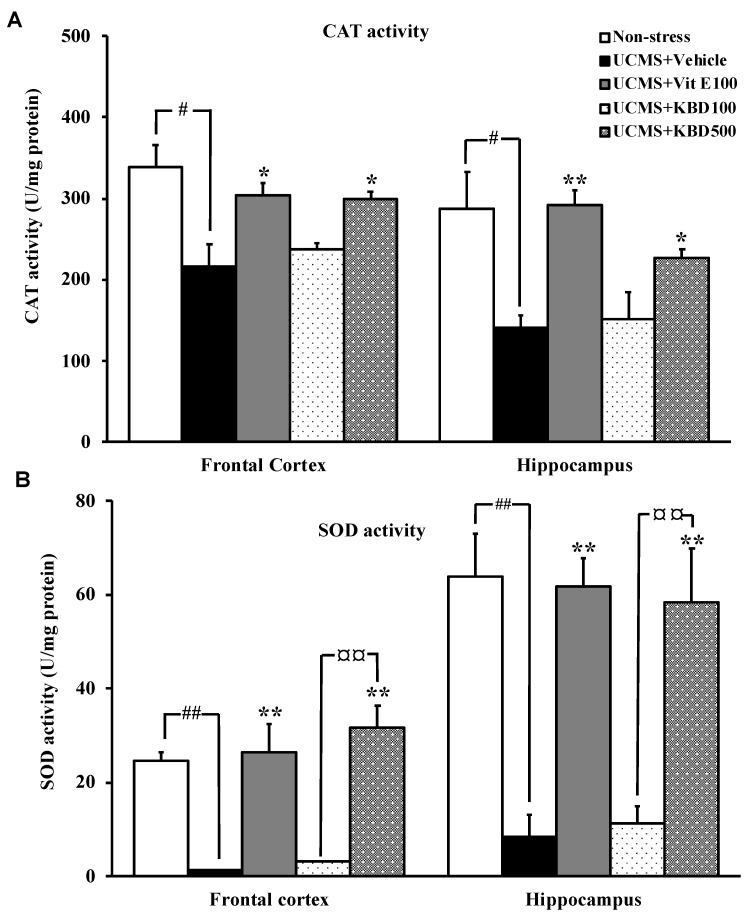
The effect of KBD and vitamin E on the activities of the brain antioxidant enzymes in the UCMS mice (**A**: CAT activity, **B**: SOD activity). Each data column represents the mean ± S.E.M (n = 3–5). ^#^*p* < 0.05 and ^##^*p* < 0.001 vs. the vehicle-treated non-stress group. ^*^ *p* < 0.05 and ^**^ *p* < 0.001 vs. the vehicle-treated UCMS group. ^¤¤^ *p* < 0.001 compared between doses of KBD.

As shown in Figure 4A,B, SOD and CAT activities were significantly decreased in the UCMS mice when compared with the non-stress control mice. Daily administration of KBD (500 mg/kg) for 3 weeks reversed the effect of the UCMS-induced reduction of the activities of the brain antioxidant enzymes in a dose-dependent manner. Treatment with vitamin E (reference antioxidant, at 100 mg/kg) also exhibited the neuroprotection by enhancing SOD and CAT activities in the frontal cortex and hippocampus of the UCMS mice.

### 2.5. Total Phenolic and Flavonoid Contents

The total phenolic and total flavonoid contents in the KBD extract and in each plant extract were calculated from the calibration curve (r^2^ = 0.9985 and r^2^ = 1, respectively). Table 1 showed the results of the total phenolic and total flavonoid contents, expressed in terms of gallic acid equivalent (GAE) and quercetin equivalent (QE), respectively. 

### 2.6. High Performance Liquid Chromatography (HPLC) Analysis of the Constituents of the KBD Extract and the Validation Method

**Figure 5:**
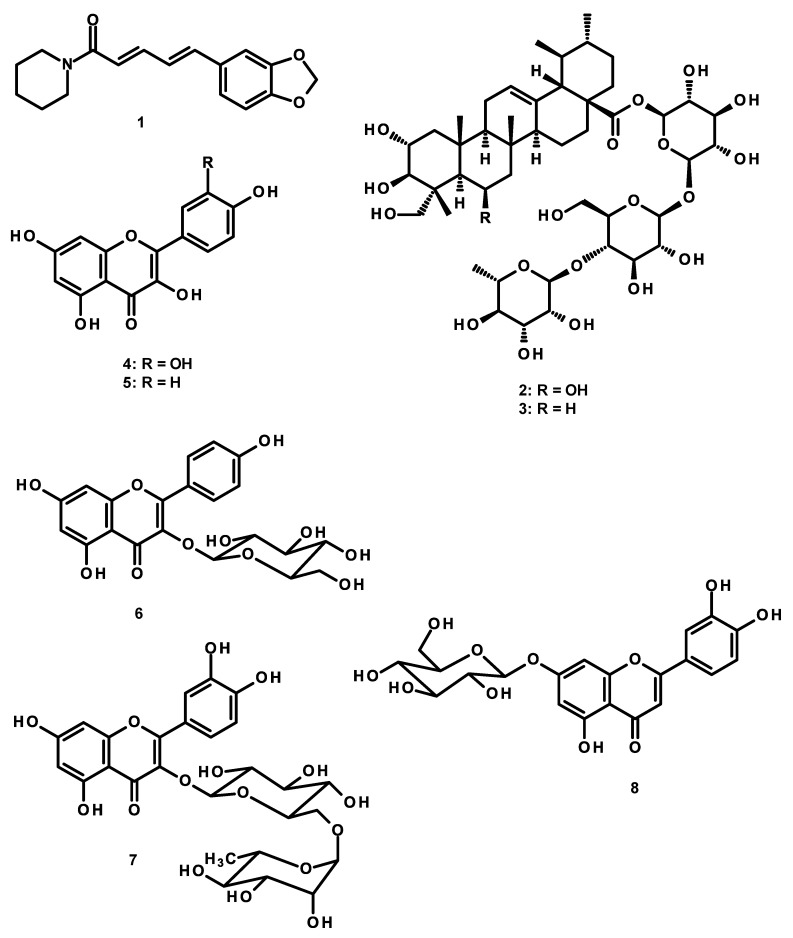
Structure of piperine (**1**), madecassoside (**2**), asiaticoside (**3**), quercetin (**4**), kaempferol (**5**), kaempferol-3-glucoside (**6**), rutin (**7**), luteolin-7-*O*-glucoside (**8**) and ferulic acid (**9**).

Piperine (**1**) (a major constituent of *P. Nigrum)*, madecassoside (**2**), and asiaticoside (**3**) (major active components of *C. asiatica*), quercetin (**4**), kaempferol (**5**), kaempferol-3-glucoside (**6**), rutin (**7**), luteolin-7-*O*-glucoside (**8**), ferulic acid (**9**) (major constituents of *N. nucifera*) were used as markers in the HPLC analysis of the KBD extract (Figure 5). Six different concentrations of standard solutions, i.e., 5–100 µg/mL for piperine (**1**), 5–30 µg/mL for madecassoside (**2**) and asiaticoside (**3**), 1–6 µg/mL for ferulic acid (**9**), luteolin-7-*O*-glucoside (**8**), rutin (**7**), kaempferol-3-glucoside (**6**), quercetin (**4**), and kaempferol (**5**), were used. This HPLC method for the analysis of these compounds was accurate and reliable, as demonstrated by the validation results. Validation parameters of the HPLC method, including range, linearity, limit of detection (LOD), limit of quantitation (LOQ), precision, and accuracy, were determined. The validation results (Supplementary Materials, Table S11) showed a good reliability of the HPLC method for the analysis of the nine compounds in the KBD extract, as demonstrated by a good linearity with the coefficient of determination, r^2^ > 0.99, appropriate percentage recoveries were always between 90–110% and the precision with low percentage relative standard deviation. This HPLC method was also used to further determine the amount of piperine (**1**) in the KBD extract, and the retention time shown in Figure S1 (Supplementary Materials) was consistent with the standard solutions (Supplementary Materials, Figure S1A) and the KBD extract (Supplementary Materials, Figure S1B). The amount of piperine (**1**) was 10.20 ± 1.081 mg/g extract. The HPLC chromatograms of the standard madecassoside (**2**) and asiaticoside (**3**) are shown in Figure S2A,B (Supplementary Materials), respectively. The amounts of madecassoside (**2**) and asiaticoside (**3**) found in the KBD extract were 179.35 ± 4.578 and 57.76 ± 3.003 mg/g extract, respectively. The HPLC chromatograms of ferulic acid (**9**), luteolin-7-*O*-glucoside (**8**), rutin (**7**), kaempferol-3-glucoside (**6**), quercetin (**4**), and kaempferol (**5**) in the KBD extract are shown in Figure S3A,B (Supplementary Materials), respectively. The amounts of ferulic acid (**9**), luteolin-7-O-glucoside (**8**), rutin (**7**), kaempferol-3-glucoside (**6**), quercetin (**4**), and kaempferol (**5**) in the KBD extract were 1.75 ± 0.128, 1.59 ± 0.464, 1.66 ± 0.289, 2.92 ± 0.063, 0.45 ± 0.039, and 0.89 ± 0.060 mg/g extract, respectively.

## 3. Discussion

The present study demonstrated that UCMS produced cognitive impairment, elevation of serum CORT, and oxidative stress in the brain. Because of the multi-pathogenesis of the UCMS-induced cognitive deficit, the classical approach modulating only at one target may not be appropriate. Therefore, a search for candidates acting at multiple targets of pathologic cascade has become an efficient strategy for drug discovery [17,18]. Treatment with KBD, a Thai traditional herbal formula consisting of three medicinal plants, was found to ameliorate UCMS-induced cognitive deficits, HPA-axis hyperactivation, and brain oxidative damage, suggesting that KBD acted on a wide range of targets associated with pathologic cascade. These findings suggested that KBD is beneficial for cognitive impairment, induced by chronic stress.

Since chronic stress is an etiological factor in cognitive impairment, the UCMS mice model is used to demonstrate the development and progress of clinical dementia [9,10]. In this study, the effect of KBD was tested in the UCMS-impaired cognition using two paradigms, indicative of different forms of memory. Spontaneous alternation, a measure of spatial working memory, is assessed by allowing mice to explore all the three arms of the Y-maze. A mouse with intact working memory, and hence intact prefrontal cortical functions, will remember the arms previously visited and shows a tendency to enter a less recently visited arm [19]. The NORT is a test for a recognition memory, which is based on a spontaneous preference for a new identical object. The choice to explore the novel object reflects the learning and recognition memory [20]. Consistent with previous studies, the UCMS mice exhibited the reduced percentage in spontaneous alternation and the less exploring time in the novel object than in the familiar one when compared with non-stress control mice. Most importantly, treatment with KBD prevented these cognition deficits, as shown by the increased percentage in spontaneous alternation and discrimination index, indicating that KBD ameliorated UCMS-induced deficits in the cognitive performance.

In order to better understand the mechanism of KBD, serum CORT levels and oxidative brain indices were investigated. It is well recognized that a primary response to prepare the body to cope with the stressful situation is the activation of the HPA axis which results in a release of CORT [9]. The negative feedback of the HPA axis is regulated by glucocorticoid receptors (GRs) in the hippocampal pyramidal cells. Chronic stress persistently impairs negative feedback mechanism, resulting in oversecretion of CORT [21]. Previous studies showed that chronically increased CORT level was associated with a reduction in hippocampal volume, and a decrease in the number of new granule cells in the dentate gyrus and pyramidal neurons in the hippocampus. These neuronal losses and degeneration may have manifested their effects as cognitive decline [22,23]. Consistent with the previous findings, we demonstrated that the serum concentration of CORT in the UCMS mice increased significantly compared to the non-stress mice. Treatment with KBD significantly prevented the elevation of serum CORT levels. This finding suggested that the protective effects of KBD on the cognitive function were accompanied by attenuating the elevation of the serum CORT.

It is widely accepted that exposure to the chronic stress causes the overproduction of free radicals and the suppression of endogenous antioxidant enzymes, which are associated with the cognitive dysfunction [24,25]. In agreement with previous reports [26,27], our present data showed that the amounts of MDA, a biomarker of lipid peroxidation, were significantly increased in the frontal cortex and hippocampus of the UCMS mice. In addition, SOD and CAT were significantly decreased in the UCMS mice when compared to the non-stress mice. Decreased activities of SOD and CAT were associated with the accumulation of these highly reactive free radicals, leading to deleterious effects such as loss of cell membrane integrity and membrane function [28]. Besides, it has been extensively reported that the oversecretion of glucocorticoids (GCs) may trigger the cellular redox system [29,30]. GRs also form a complex with B-cell lymphoma 2 (Bcl-2) and translocate into mitochondria in response to CORT and modulate mitochondrial oxidation. The enhancement of cellular metabolic rate promotes ATP synthesis leading to a spontaneous superoxide anion (O**^.-^**_2_) generation via mitochondrial complexes I and III of the electron transport chain. An increase in the production of O**^.-^**_2_ and other radicals, with concurrent inhibition of the endogenous antioxidant defense mechanism, ultimately resulted in neuronal cell death, especially in the pyramidal cell of the hippocampus where GRs are located and thus causing further cognitive decline [31]. Interestingly, KBD significantly improved the brain oxidative status in the UCMS mice, as demonstrated by reduced MDA level and elevated activities of antioxidant enzymes, which is similar to the effects of vitamin E. Moreover, the determination of total phenolic and flavonoid contents showed that the KBD extract contained high total phenolic (121.710 ± 3.547 mg GAE/g extract) and total flavonoid (70.523 ± 0.007 mg QE/g extract) contents, thus supporting the antioxidative property of the KBD. Based on the obtained data, it can be inferred that the neuroprotective effect of KBD is similar to that of vitamin E. It is important to point out that the UCMS model produces not only cognitive impairment but also depression with various neuropathologies such as reduction of neurogenesis, neuroinflammation, and neurotransmitter changes [10]. Although the neuroprotective effect of KBD is found to be similar to that of vitamin E in the present study, their modes of action are different. It is well-known that pharmacological activity of vitamin E is related to its lipid solubility; however, its antidepressive effect is still controversial. On the other hand, the antidepressive activity of *P. nigrum, N. Nucifera,* and *C. asiatica* is related to the anti-inflammatory effect and the enhancement of neurogenesis [32,33,34]. Therefore, KBD may have more beneficial effects than vitamin E.

Meanwhile, phytochemical analysis of the KBD extract by HPLC fingerprint method revealed the presence of piperine (**1**), madecassoside (**2**), asiaticoside (**3**), quercetin (**4**), kaempferol (**5**), kaempferol-3-glucoside (**6**), rutin (**7**), luteolin-7-*O*-glucoside (**8**), and ferulic acid (**9**). The radical scavenging and antioxidant activities of these compounds have been extensively reported [35,36,37,38]. Moreover, accumulating evidences suggested that these dietary phytochemicals potently strengthen the antioxidative potential through the modulation of the Nrf2/antioxidant responsive element (ARE) signaling pathway [39,40,41,42]. Nrf2 is a master regulator of the antioxidant response. Upon exposure to oxidative stress, Nrf2 is activated and translocates from the cytoplasm to the nucleus where it sequentially binds to the ARE. This binding induces the expression of ARE-containing genes, including NADPH quinine oxidoreductase 1 (NQO-1), heme oxygenase 1 (HO-1), SOD, CAT, and glutathione peroxidase, which can respond quickly to oxidative stress in order to maintain the balanced redox state [43,44]. In addition, neuroinflammation, one of the main mechanisms in the UCMS-induced neuronal cell death, is closely linked to the oxidative stress and activation of the nuclear factor kappa B (NF-κB). Natural antioxidants such as flavonoids and phenolic compounds possess a strong anti-inflammatory activity, which contributes to their neuroprotective effect [44,45]. Coadministration of quercetin (**4**) and piperine (**1**) attenuated UCMS-induced increased levels of TNF-α in mice brain [46]. Kaempferol (**5**) and rutin (**7**) decreased the release of pro-inflammatory cytokines by inhibiting AKT phosphorylation and NFκB activation [47]. Taken together, the present findings suggested that the memory-enhancing effects of KBD could be due to the improvement of antioxidant capacity of the flavonoid, phenolic acid, and triterpene glycosides, which are constituents of the plant ingredients, as well as the suppression of the HPA axis hyperactivation from the chronic stress exposure. 

Because of the importance of the effect of phytochemicals on Nrf2/ARE signaling pathway, the effect of KBD on Nrf2 activation and neuroinflammation in the UCMS mice need to be further investigated to confirm the underlying mechanisms.

## 4. Experimental Section

### 4.1. Preparation of the KBD Extract 

KBD was provided by Pho-Ngern Osot, the Pharmacy of the Chao Phraya Abhaibhubate Hospital, in Prachinburi Province, Thailand. Dried powdered KBD (300 g) was macerated in 1.5 L of 95% ethanol for 3 days, at room temperature, and filtered. The process was repeated three times, and the ethanol solutions were combined and concentrated, under reduced pressure at 50 °C, and then freeze-dried to give 3.8 g of the KBD extract (11.32 yield). The extract was kept at −20 °C throughout the experiment.

### 4.2. Animal

**Figure 6:**
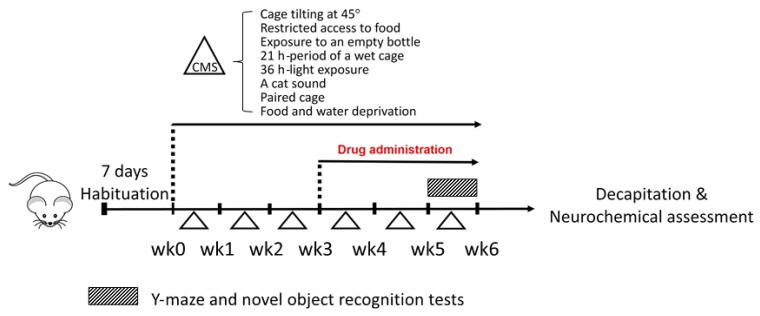
A schematic presentation of the experimental protocols. Mice were divided into a non-stress group and the chronic mild stress (UCMS) group at week 0. The UCMS group was exposed to various unpredictable, stressful stimuli from week 0 to week 6. The UCMS group was divided into four groups which were daily administered with (i) vehicle, (ii) vitamin E (100 mg/kg, p.o.), (iii) KBD (100 mg/kg, p.o), and (iv) KBD (500 mg/kg, p.o) for 3 weeks after day 21. Y-maze test and NORT were conducted at week 5 to week 6. Twenty-four hours after the behavioral test, the animals were decapitated to collect the blood and brain for neurochemical assessment.

This study was conducted according to the experimental protocols described in Figure 6. Male ICR mice (20–30 g, 5 weeks old) were obtained from the Nomura Siam International Company (Bangkok, Thailand). Mice were housed in wood chip bedding in stainless steel cages with free access to food and water. Housing was thermostatically maintaining at 22 ± 2 °C with constant humidity (45% ± 2%) and a 12-h light-dark cycle (light on 06:00–18:00) in the Laboratory Animal Unit of the Faculty of Pharmaceutical Sciences, Khon Kaen University, Thailand. All behavioral tests were performed from 07:30 to 17:00 h. The experimental protocols used in the present study were in accordance with the Guiding Principles for the Care and Use of Animal (NIH Publications #8-23, revised in 2011) and were also approved by Animal Ethics Committee for Use and Care of Khon Kaen University, Khon Kaen, Thailand (approval No. IACUC-KKU-36/61).

### 4.3. Unpredictable Chronic Mild Stress (UCMS)

The UCMS model was used to induce depressive-like symptoms in mice, as previously described by Daodee et al. [48]. Mice were randomly divided into five groups; a non-stress group (control group) and four other groups, subjected to UCMS for 6 weeks. The UCMS procedures consisted of a variety of stressors including one period of food and water deprivation (18 h), two periods of tilted cage at 45° (12 h), two periods of restricted access to food of five micro pellets (1 h), two periods of exposure to empty bottle (3 h), one period of wet cage (21 h), two periods of light exposure (36 h), two periods of cat sound (3 h, 5 h), and two periods of paired caging (2 h). All of these stressors were randomly scheduled over one week in day-night time and repeated for 6 weeks of the experiment as shown in Figure 6. The non-stress control mice were housed under normal environments.

### 4.4. Drug Administration

Vehicle (0.5% sodium carboxymethyl cellulose, SCMC, HiMedia laboratories, Mumbai, India), vitamin E (Sigma-Aldrich, St. Louis, MO, USA) and KBD were daily administered for 3 weeks after day 21 (Figure 6) at 8.00 am, as previously described by Daodee et al. [48]. On the behavioral testing day, all treatments were conducted 1 h before testing. The mice were divided into five groups (n = 10–12). The first group was a non-stress control group that received 0.5% SCMC (1 mL/kg, p.o.) (n = 8). The second group was the UCMS group which received 0.5% SCMC (1 mL/kg, p.o.) (n = 10). The third group was the UCMS group which received vitamin E (100 mg/kg, p.o.) (n = 10). The fourth and fifth groups were the UCMS groups which received KBD 100 mg/kg, p.o (n = 8) and 500 mg/kg, p.o (n = 8), respectively. The clinical dose of KBD, which is prescribed in the hospital, is 2000 mg/day. This dose was converted into the appropriate dose for mice, according to the following equation: human equivalent dose (HED, mg/kg) = mouse dose (mg/kg) x (mouse K_m_/Human K_m_), where K_m_ is the correction factor [49]. For example, if the human average weight is 60 kg, the clinical equivalent dose calculated for mouse is (2000/60) × 37/2.85 = 432.75 mg/kg/day. Thus, the dose of 500 mg/kg/day was used as the effective dose and the dose of 100 mg/kg/day was used as an ineffective dose for KBD. KBD (1 g) was freshly suspended in 0.5% SCMC (10 mL) and used as a stock solution. Twenty-four hours after the behavioral test, the mice were decapitated, and the serum, frontal cortex, and hippocampus were collected immediately and kept at −80 °C throughout the experiment.

### 4.5. Behavioral Analysis

#### 4.5.1. Y-Maze Test

The Y-maze test consists of three arms of equal size of 40 cm long, 3 cm wide at the bottom and 12 cm wide at the top, and oriented at 60° angle from each tail. This apparatus was used to assess a spontaneous alternation. The number and sequence of arm entry were observed for 8 minutes. Alternation was defined as successive entries into three different arms, e.g., 123, 321, 132, 213, but not 323. The percentage of alternation was calculated according to the equation:

% Alternation = (Number of alternations/Total arm entries-2) × 100

#### 4.5.2. Novel Object Recognition Test (NORT)

The novel object recognition test (NORT) was used to investigate the memory working behavior as previously described [20]. The NORT consisted of three sessions: habituation, sample phase trial, and test phase trial. Mice were placed in a plastic square arena (50 cm length × 50 cm width × 40 cm height). Twenty four hours before the test, each mouse was given one habituation session in the box to explore the arena without stimuli (without objects) with 10 min exploration. In the sample phase trial, each mouse was placed in the arena box where two identical objects were located in the corner at a specified distance from each other, and was allowed to explore for 5 minutes. The mouse was considered to be exploring the object when its head was facing, touching, or sniffing the object. The exploring time was recorded. The test phase trials were performed 30 min after the sample phase trials. One of the two objects was replaced by a novel object. The mouse was exposed to the objects for 5 minutes, and the total time spent exploring the familiar object and the novel object were recorded and analyzed. At the interval of the trials, the square arena and identical objects were cleaned using 70% ethanol to avoid a build-up of olfactory cues. The exploration ratio was calculated according to the equation:

% Discrimination index = {(TN − TF)/(TN + TF)} × 100

where TN and TF are the exploring times of the novel and familiar objects during 5 min observation period, respectively.

### 4.6. Determination of Serum CORT Levels 

Blood samples were collected immediately after decapitation via cardiac puncture under the anesthetic, pentobarbital sodium (Nembutal^®^: 60 mg/kg, i.p.; Ceva Sante Animale, Libourne, France). Blood samples were left at room temperature and centrifuged at 3000 rpm at 21 °C for 20 min. The serum CORT level was determined using corticosterone ELISA kit (Assaypro LLC. St. Charies, MO, USA). The CORT levels was determined according to the previously described procedure [49]. The CORT standard and sample (25 µL) were added in 96-well microplates of ELISA kit, and immediately added biotinylated corticosterone (25 µL) to each well. The microplates were mixed gently and incubated for 2 hours at room temperature. Each well was washed five times with 200 µL of a wash buffer manually, and streptavidin-peroxidase conjugated (50 µL) was ad dedto each well, and incubated for 30 min. Then, each well was washed with 200 µL of a wash buffer manually for five times, and the chromogen substrate (50 µL) was added, and incubated for 20 minutes. The reaction was stopped with the stop solution (50 µL). The microplate reader (Perkin Elmer Inc., Waltham, MA, USA) was used to measure the absorbance at 450 nm.

### 4.7. Determination of MDA Contents by the Thiobarbituric Acid Reactive Substances (TBARS) Assay

The lipid peroxidation was measured in the homogenates of the brain frontal cortex and hippocampus, and their protein contents were quantified according to the Bradford method [20]. Briefly, the frontal cortex or hippocampus was weighed and homogenized in 10 volumes of phosphate buffer (5 mM, pH 7.4). The homogenized brain was centrifuged, and the supernatant was mixed with 10% trichloroacetic acid (TCA) (Sigma-Aldrich, St. Louis, MO, USA) and centrifuged at 8000× *g*, 4 °C for 10 minutes. The supernatant was collected and incubated with 0.8% (w/v) of 2-thiobarbituric acid (TBA) (Sigma-Aldrich, St. Louis, MO, USA) at 100 °C for 15 min. The intensity of a pink pigment formed from the MDA-TBA condensation indicates the extent of lipid peroxidation. The complexes were quantified using the UV/visible spectrophotometer at 532 nm. MDA (Sigma-Aldrich, St. Louis, MO, USA) was used as a standard. The results were represented as pmol of MDA/mg protein.

### 4.8. Determination of Catalase (CAT) and Superoxide Dismutase (SOD) Activities

The activities of SOD and CAT in the hippocampus and frontal cortex homogenates were determined using commercially available kits from Sigma-Aldrich (St. Louis, MO, USA). All the data of antioxidant enzymes activities, brain MDA and oxidative index levels were normalized to their total protein concentration in the sample in order to account for possible differences in protein concentrations between samples. The protein concentration of the hippocampus and frontal cortex homogenates was determined by the Bradford method [48].

### 4.9. Determination of Total Phenolic and Flavonoid Contents

The total phenolic content was determined using the Folin-Ciocalteu assay, as described previously [50]. Twenty µL of the KBD, *P. nigurm, C. asiatica,* and *N. nucifera* extracts were added into a 96-well plate, followed by100 µL of 10% Folin-Ciocalteu reagent (Sigma-Aldrich, St. Louis, MO, USA) and 80 µL of 7% sodium carbonate. The solutions were mixed and kept from light for 30 minutes, and the absorbance was measured at 760 nm by a microplate reader (Perkin Elmer Inc., Waltham, MA, USA). Total phenolic content was expressed as mg gallic acid (Sigma-Aldrich, St. Louis, MO, USA) equivalents (GAE) per gram of extract. The total flavonoid content was determined using the aluminum chloride colorimetric method. Twenty µL of each extract was added into a 96-well plate and mixed with 15 µL of aluminum chloride, 20 µL of 10% sodium acetate and 145 µL of distilled water. The solutions were incubated and protected from light for 15 min. The absorbance was then measured at 430 nm. The total flavonoid content was expressed as mg quercetin (Sigma-Aldrich, St. Louis, MO, USA) equivalents (QE) per gram of extract. 

### 4.10. HPLC Analysis and Validation of the Analytical Method 

The KBD extract was analyzed by a reversed-phase HPLC system equipped with a UV detector (Agilent Technologies Inc., Santa Clara, CA, USA), using a Hypersil ODS column (4 × 250 mm, 5 µm) for the analysis of madecassoside (**2**), asiaticoside (**3**), ferulic acid (**9**), luteolin-7-*O*-glucoside (**8**), rutin (**7**), kaempferol-3-glucoside (**6**), quercetin (**4**), and kaempferol (**5**), while piperine (**1**) was analyzed by another reversed-phase HPLC system (Shimadzu USA Manufacturing Inc., Japan), using the same column. Methanol (Solvent A) and 0.2% formic acid in aqueous solution (solvent B) were used as mobile phases in the analysis of ferulic acid (**6**), luteolin-7-*O*-glucoside (**8**), rutin (**7**), kaempferol-3-glucoside (**6**), quercetin (**4**) and kaempferol (**5**), using a gradient elution of 30–60% solvent A during 65 min with a flow rate of 1.2 mL/min, and detected at 254, 275, and 370 nm. The HPLC analysis of madecassoside (**2**) and asiaticoside (**3**) was performed by a gradient elution with 20–50% of acetonitrile and ultrapure water with a flow rate of 1 mL/min, and detected at 206 nm. For the analysis of piperine (**1**), the mobile phase consisted of 1% formic acid: 2-propanol: acetonitrile (55: 10: 35, v/v/v), using isocratic elution with a flow rate of 1.2 mL/min, and detected at 254 nm.

The amount of ferulic acid (**9**), luteolin-7-*O*-glucoside (**8**), rutin (**7**), kaempferol-3-glucoside (**6**), quercetin (**4**), and kaempferol (**5**) present in the KBD extract was then determined by comparison with standard curves (1, 2, 3, 4, 5, 6 µg/mL). The standard solutions of madecassoside (**2**) and asiaticoside (**3**), at 5, 10, 15, 20, 25, and 30 µg/mL, were prepared and used to establish the calibration curve from the stock solution of 1 mg/mL. Six standard solutions of piperine (**1**), at 5, 10, 25, 50, 75, and 100 µg/mL, were also prepared by dilution of a stock solution of 1 mg/mL with methanol. The analytical method was validated for specificity by the absence of undesired peaks in HPLC chromatograms and for accuracy by the percentage recovery of all standards in 5 replicates. Precision (% RSD) was validated for within-day and between-day determinations (n = 5). Linearity was validated by using linear regression analysis to calculate the coefficient of determination (r^2^) of the standard curve (n = 5). Limit of detection (LOD) and limit of quantitation (LOQ) were also determined, and signal to noise ratios were calculated (n = 5).

### 4.11. Statistic Analysis

All data were expressed as mean ± S.E.M. The analysis was performed by paired Student’s *t*-test for two groups or one-way analysis of (ANOVA) variance, followed by the Tukey test for multiple comparisons among different groups. The difference with *p* < 0.05 is considered significant. The analysis was conducted using SigmaStat^®^ ver. 3.5 (SYSTAT variance Inc., Richmond, CA, USA).

## 5. Conclusions

The present investigation demonstrated that a chronic stress exposure induced memory and cognitive decline, which is due to the stress-mediated HPA axis hyperactivation and oxidative brain damage. Daily treatment with the KBD extract was found to improve cognitive impairment in the UCMS mice. Notably, the therapeutic effects of KBD mainly involved the reduction of serum CORT level as well as the neuroprotective effect against the UCMS-induced neuronal oxidative damage by decreasing lipid peroxidation and increasing endogenous antioxidant enzymes, e.g., SOD and CAT, activities. Phytochemical analysis of the extracts of KBD and the individual plant components revealed that the effects of KBD could be due to, in part, the pharmacological activities of phenolics, flavonoids and triterpene glycosides, which are major constituents of the three plant ingredients of this Thai herbal remedy. Thus, the results of this study warrant the use of KBD as an effective formula for daily stress life events related to learning and memory deficit.

## Figures and Tables

**Table 1:** Total phenolic and total flavonoid contents of the KBD extract and each component extracts.

Extract	Total Phenolic Content (mg GAE/g extract)	Total Flavonoid Content (mg QE/g Extract)
*Piper nigrum*	95.234 ± 0.008	77.837 ± 0.011
*Centella asiatica*	51.226 ± 0.015	22.709 ± 0.001
*Nelumbo nucifera*	207.195 ± 0.022	54.612 ± 0.010
KBD	121.710 ± 3.547	70.523 ± 0.007

## Supplementary Materials

The following are available online. Table S1. One-way analysis of various (ANOVA) tests of the Y-maze test. Table S2. One-way analysis of variance (ANOVA) test of novel object recognition test (NORT). Table S3. Time exploring object (compared with familiar object). Table S4. One-way analysis of variance (ANOVA) test of serum corticosterone (CORT) level. Table S5. One-way analysis of variance (ANOVA) test of TBARS assay (Frontal cortex). Table S6. One-way analysis of various (ANOVA) test of TBARS assay (hippocampus).Table S7. One-way analysis of various (ANOVA) test of catalase activity (frontal cortex). Table S8. One-way analysis of various (ANOVA) test of catalase activity (hippocampus). Table S9. One-way analysis of various (ANOVA) test of superoxide dismutase activity (Frontal cortex). Table S10. One-way analysis of various (ANOVA) test of superoxide dismutase activity (hippocampus). Table S11. Validation results of the analytical method for the determination of piperine, madecassoside, asiaticoside, ferulic acid, luteolin-7-*O*-glucoside, rutin, kaempferol-3-glucoside, quercetin, and kaempferol content in the KBD extract. Figure S1. HPLC chromatograms of piperine solution (**A**) and the KBD extract (**B**). Figure S2. HPLC chromatograms of madecassosside and asiaticoside solution (**A**) and the KBD extract (B). Figure S3. HPLC chromatograms of six standards*’* solution (**A**) and the KBD extract (**B**) (1 *=* Ferulic acid, 2 *=* Luteolin*-*7*-*O*-*glucoside, 3 *=* Rutin, 4 *=* Kaempferol*-*3*-*glucoside, 5 *=* Quercetin, 6 *=* Kaempferol).
