# Supplementary material for: Kleeb Bua Daeng, a Thai Traditional Herbal Formula, Ameliorated Unpredictable Chronic Mild Stress-Induced Cognitive Impairment in ICR Mice
Source: Molecules. 2019 Dec 14;24(24):4587. doi: 10.3390/molecules24244587 (PMC6943511; doi:10.3390/molecules24244587)
Supplement: Supplementary file 1 [file molecules-24-04587-s001.pdf]

Supplementary Materials

Kleeb Bua Daeng, a Thai Traditional Herbal Formula, Ameliorated Unpredictable Chronic Mild Stress-Induced Cognitive Impairment in ICR Mice

Juthamart Maneenet [1,2], Supawadee Daodee [2], Orawan Monthakantirat [2], Chantana Boonyarat [2], Charinya Khamphukdee [2], Pakakrong Kwankhao [3], Supaporn Pitiporn [3], Suresh Awale [4], Yaowared Chulikhit [2,*] and Anake Kijjoa [5,*]

[1] Graduate School of Pharmaceutical Sciences, Khon Kaen University, Khon Kaen 40002, Thailand; juthamart_pp@hotmail.com (J.M.)

[2] Division of Pharmaceutical Chemistry, Faculty of Pharmaceutical Sciences, Khon Kaen University, Khon Kaen 40002, Thailand; csupawad@kku.ac.th (S.D.); oramon@kku.ac.th (O.M.); chaboo@kku.ac.th (C.B.); charkh@kku.ac.th (C.K.)

[3] Department of Pharmacy, Chao Phya Abhaibhubejhr Hospital, Ministry of Public Health, Prachinburi, Thailand 25000, Thailand; pakakrong2@gmail.com (P.K.); spitiporn@yahoo.com (S.P.)

[4] Division of Natural Drug Discovery, Institute of Natural Medicine, University of Toyama, 2630 Sugitani, Toyama 930-0194, Japan; suresh@inm.u-toyama.ac.jp

[5] *ICBAS*-Instituto de Ciências Biomédicas Abel Salazar and CIIMAR, Rua de Jorge Viterbo Ferreira, 228, 4050-313 Porto, Portugal

* Correspondence: yaosum@kku.ac.th (Y.C.); ankijjoa@icbas.up.pt (A.K.), Tel.: +351-22042-8331 (A.K.)

1. ***Statistical analysis of KBD on Unpredictable Chronic Mild Stress (UCMS)-Induced Cognitive Impairment***.

Table S1. One-way analysis of variance (ANOVA) test of the Y-maze test.

Group comparison	ANOVA followed by Tukey's post hoc test	
	P	*F* ($DF_{between\ group}$, $DF_{residual}$)
non-stress group VS. vehicle-treated UCMS group	<0.001	$F_{(4,43)}$=8.973
vehicle-treated UCMS group VS. UCMS+Vit E100	0.019	
vehicle-treated UCMS group VS. UCMS+KBD100	0.121	
vehicle-treated UCMS group VS. UCMS+KBD500	<0.001	
UCMS+KBD100 VS. UCMS+KBD500	0.015	

Table S2. One-way analysis of variance (ANOVA) of the Novel Object Recognition Test (NORT).

Group comparison	ANOVA followed by Tukey's post hoc test	
	P	*F* ($DF_{between\ group}$, $DF_{residual}$)
non-stress group VS. vehicle-treated UCMS group	0.002	$F_{(4,41)}$=7.77
vehicle-treated UCMS group VS. UCMS+Vit E100	0.002	
vehicle-treated UCMS group VS. UCMS+KBD100	0.006	
vehicle-treated UCMS group VS. UCMS+KBD500	<0.001	

Table S3. T-test of time exploring object (comparison between the new object and the familiar object).

Group comparison	*t*-test		
	t	Df	*P*
non-stress group (familiar) VS. non-stress group (new)	-16.299	-26.299	<0.001
vehicle-treated UCMS group (familiar) VS. vehicle-treated UCMS group (new)	-1.046	-4.261	0.313
UCMS+Vit E100 (familiar) VS. UCMS+Vit E100 (new)	-11.486	-25.471	<0.001
UCMS+KBD100 (familiar) VS. UCMS+KBD100 (new)	-2.925	-6.689	0.011
UCMS+KBD500 (familiar) VS. UCMS+KBD500 (new)	-4.595	-18.031	<0.001

2. ***Statistical analysis of the KBD extract on UCMS-Induced hypersecretion of serum corticosterone***.

Table S4. One-way analysis of variance (ANOVA) test of serum corticosterone (CORT) levels.

Group comparison	ANOVA followed by Tukey's post hoc test	
	P	*F* ($DF_{between\ group}$, $DF_{residual}$)
non-stress group VS. vehicle-treated UCMS group	<0.001	$F_{(4,10)}$=16.047
vehicle-treated UCMS group VS. UCMS+Vit E100	0.003	
vehicle-treated UCMS group VS. UCMS+KBD100	0.004	
vehicle-treated UCMS group VS. UCMS+KBD500	<0.001	

3. ***Statistical analysis of KBD on the UCMS-Induced lipid peroxidation***.

Table S5. One-way analysis of variance (ANOVA) test of the lipid peroxidation in the frontal cortex.

Group comparison	ANOVA followed by Tukey's post hoc test	
	P	F ($DF_{between\ group}$, $DF_{residual}$)
non-stress group VS. vehicle-treated UCMS group	<0.001	$F_{(4,17)}$=525.556
vehicle-treated UCMS group VS. UCMS+Vit E100	<0.001	
vehicle-treated UCMS group VS. UCMS+KBD100	<0.001	
vehicle-treated UCMS group VS. UCMS+KBD500	<0.001	

Table S6. One-way analysis of variance (ANOVA) test of lipid peroxidation in the hippocampus.

Group comparison	ANOVA followed by Tukey's post hoc test	
	P	F ($DF_{between\ group}$, $DF_{residual}$)
non-stress group VS. vehicle-treated UCMS group	<0.001	$F_{(4,16)}$=32.222
vehicle-treated UCMS group VS. UCMS+Vit E100	<0.001	
vehicle-treated UCMS group VS. UCMS+KBD100	<0.001	
vehicle-treated UCMS group VS. UCMS+KBD500	<0.001	

4. ***Statistical analysis of KBD on the UCMS-Induced Oxidative Stress in the Brain***.

Table S7. One-way analysis of variance (ANOVA) test of the catalase activitiy in the frontal cortex

Group comparison	ANOVA followed by Tukey's post hoc test	
	P	F ($DF_{between\ group}$, $DF_{residual}$)
non-stress group VS. vehicle-treated UCMS group	0.006	$F_{(4,14)}$=6.275
vehicle-treated UCMS group VS. UCMS+Vit E100	0.046	
vehicle-treated UCMS group VS. UCMS+KBD100	0.99	
vehicle-treated UCMS group VS. UCMS+KBD500	0.048	

Table S8. One-way analysis of variance (ANOVA) test of the catalase activitiy in the hippocampus

Group comparison	ANOVA followed by Tukey's post hoc test	
	P	F ($DF_{between\ group}$, $DF_{residual}$)
non-stress group VS. vehicle-treated UCMS group	0.02	$F_{(4,13)}$=7.538
vehicle-treated UCMS group VS. UCMS+Vit E100	<0.001	
vehicle-treated UCMS group VS. UCMS+KBD100	0.998	
vehicle-treated UCMS group VS. UCMS+KBD500	0.004	

Table S9. One-way analysis of variance (ANOVA) test of the superoxide dismutase (SOD) activity in the frontal cortex.

Group comparison	ANOVA followed by Tukey's post hoc test	
	P	*F* ($DF_{between\ group}$, $DF_{residual}$)
non-stress group VS. vehicle-treated UCMS group	<0.001	$F_{(4,14)}$=17.385
vehicle-treated UCMS group VS. UCMS+Vit E100	<0.001	
vehicle-treated UCMS group VS. UCMS+KBD100	0.996	
vehicle-treated UCMS group VS. UCMS+KBD500	<0.001	
UCMS+KBD100 VS. UCMS+KBD500	<0.001	

Table S10. One-way analysis of variance (ANOVA) test of the SOD activity in the hippocampus.

Group comparison	ANOVA followed by Tukey's post hoc test	
	P	*F* ($DF_{between\ group}$, $DF_{residual}$)
non-stress group VS. vehicle-treated UCMS group	<0.001	$F_{(4,15)}$=21.732
vehicle-treated UCMS group VS. UCMS+Vit E100	<0.001	
vehicle-treated UCMS group VS. UCMS+KBD100	0.997	
vehicle-treated UCMS group VS. UCMS+KBD500	<0.001	
UCMS+KBD100 VS. UCMS+KBD500	<0.001	

Table S11. Validation results of the analytical method for determination of piperine (**1**), madecassoside (**2**), asiaticoside (**3**), ferulic acid (**9**), lutiolin-7-*O*-glucoside (**8**), rutin (**7**), kaempferol-3-glucoside (**6**), quercetin (**4**) and kaempferol (**5**) content in the KBD extract.

Parameter		Compounds								
		**Piperine**	**Madecassoside**	**Asiaticoside**	**Ferulic acid**	**Luteolin-7-*O*-glucoside**	**Rutin**	**Kaempferol-3-glucoside**	**Quercetin**	**Kaempferol**
**Linearity**	**Range (µg/mL)**	5-100	5-30	5-30	1-6	1-6	1-6	1-6	1-6	1-6
	**Coefficient Determination (R^2)**	0.9994 ± 0.002	0.9937 ± 0.001	0.9917 ± 0.004	0.9984±0.001	0.9988±0.001	0.9951±0.002	0.9958±0.003	0.9901±0.001	0.9938±0.005
**LOD (µg/mL)**		0.05 (S/N ~ 3.417 ± 0.545)	2.5 (S/N ~ 2.814 ± 0.986)	2.5 (S/N ~ 3.129 ± 0.150)	0.5 (S/N~3.206 ± 0.714)	0.5 (S/N~4.284 ± 0.434)	0.5 (S/N~4.027 ± 0.348)	0.5 (S/N~4.453 ± 1.240)	0.5 (S/N~3.976 ± 0.551)	0.5 (S/N~3.814 ± 0.277)
**LOQ (µg/mL)**		0.1 (S/N ~ 10.175 ± 2.300)	5 (S/N ~ 10.643 ± 0.098)	5 (S/N ~ 10.400 ± 0.129)	1 (S/N~11.051 ± 1.737)	1 (S/N~12.853 ± 1.303)	1 (S/N~12.086 ± 1.044)	1 (S/N~11.930 ± 2.272)	1 (S/N~11.927 ± 1.652)	1 (S/N~11.443 ± 0.826)
**Precision (%RSD)**	**Within day**	0.719 – 2.710	0.698 – 1.594	0.620 – 1.728	0.420 - 1.386	0.598 - 1.985	0.009 - 1.400	0.531 - 1.944	0.141 - 1.616	0.616 - 3.761
	**Between day**	0.828 – 5.012	0.978 – 2.700	1.110 – 3.000	1.228 - 3.464	0.749 - 5.765	1.789 - 6.725	2.197 - 5.553	2.127 - 4.123	0.299 - 4.892
**Accuracy %**	**Conc. (Low)**	96.243 ± 0.061	92.704 ± 6.252	101.436 ± 4.525	99.728 ± 0.812	100.233 ± 6.294	109.538 ± 2.776	106.655 ± 4.296	106.232 ± 4.420	104.835 ± 3.582

Recovery	Conc. (Medium)	102.091 ± 0.387	99.533 ± 2.469	97.377 ± 1.570	98.778 ± 3.529	100.429 ± 4.888	96.342 ± 3.207	98.940 ± 3.481	96.507 ± 10.665	94.153 ± 4.930
	Conc. (High)	100.693 ± 2.697	103.564 ± 3.076	97.584 ± 0.464	100.732 ± 2.034	99.324 ± 1.880	102.049 ± 1.789	101.893 ± 4.849	102.726 ± 5.254	104.734 ± 2.941

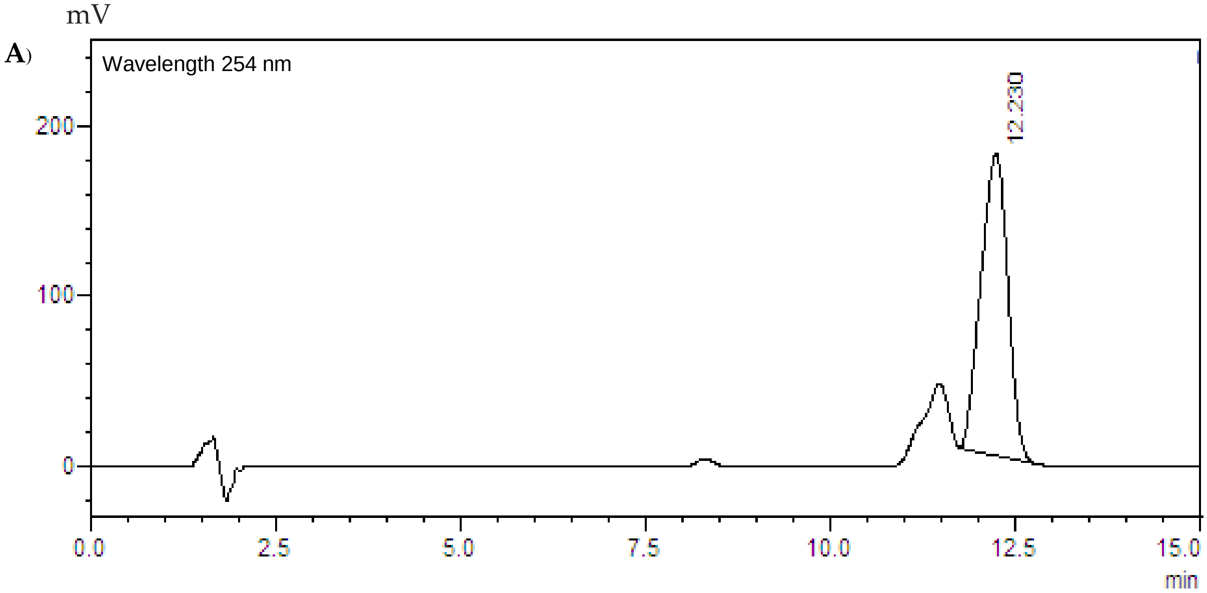

Figure S1. HPLC chromatograms of piperine solution (**A**) and the KBD extract (**B**).

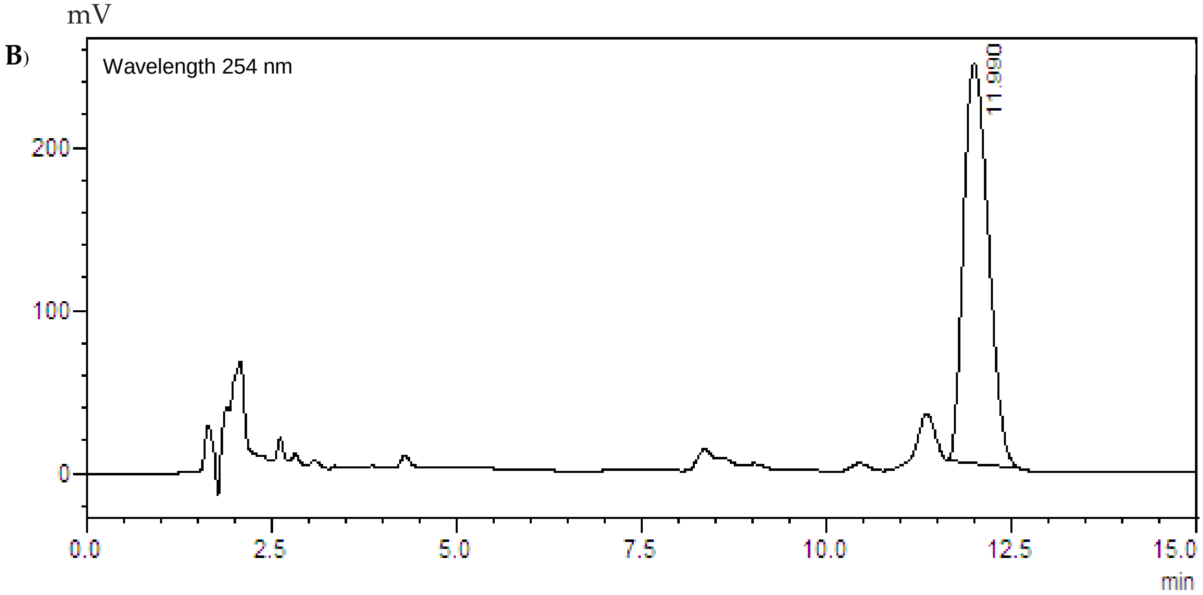

Figure S2. HPLC chromatograms of madecassosside and asiaticoside solution (A) and the KBD extract (**B**).

A)

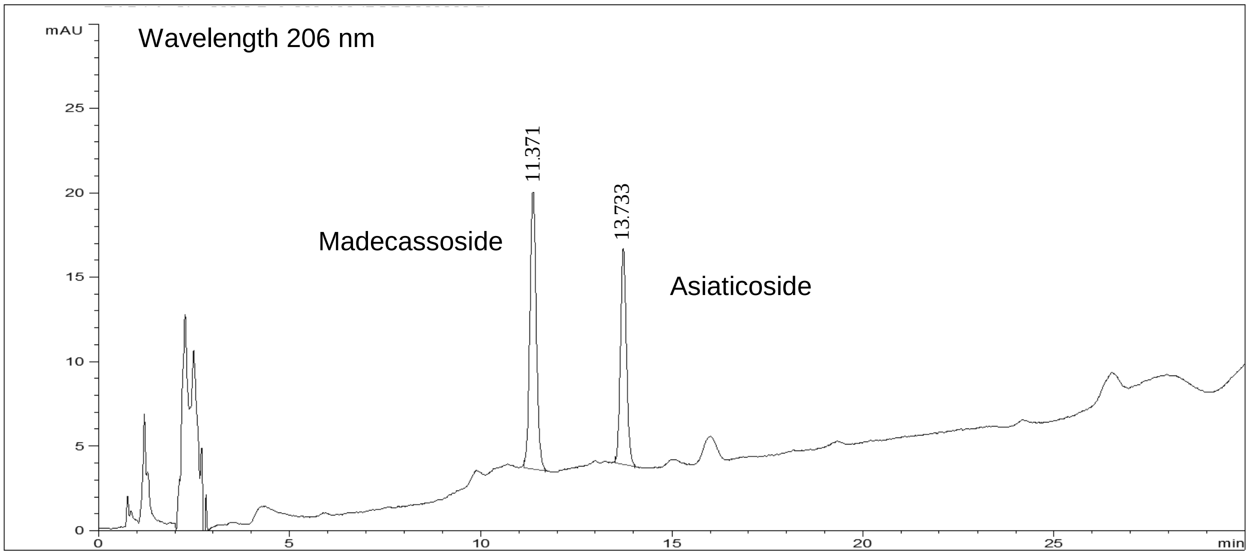

B)

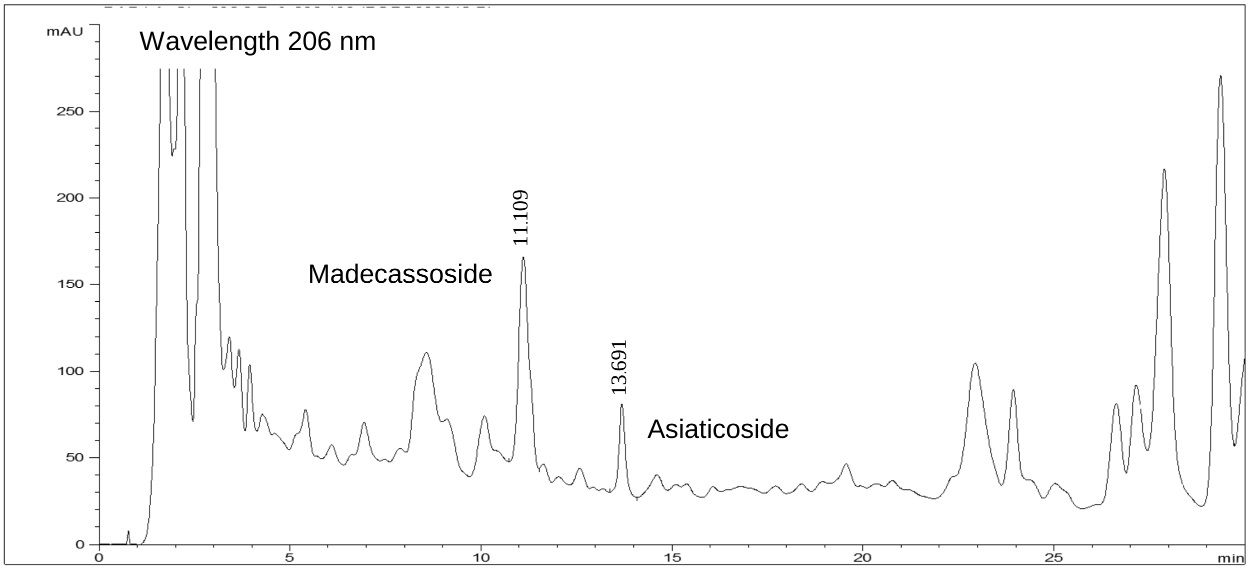

Figure S3. HPLC chromatograms of six standards' solution (**A**) and the KBD extrac (**B**) (1 = ferulic acid, 2 = luteolin-7-*O*-glucoside, 3 = rutin, 4 = kaempferol-3-glucoside, 5 = quercetin, 6 = kaempferol).

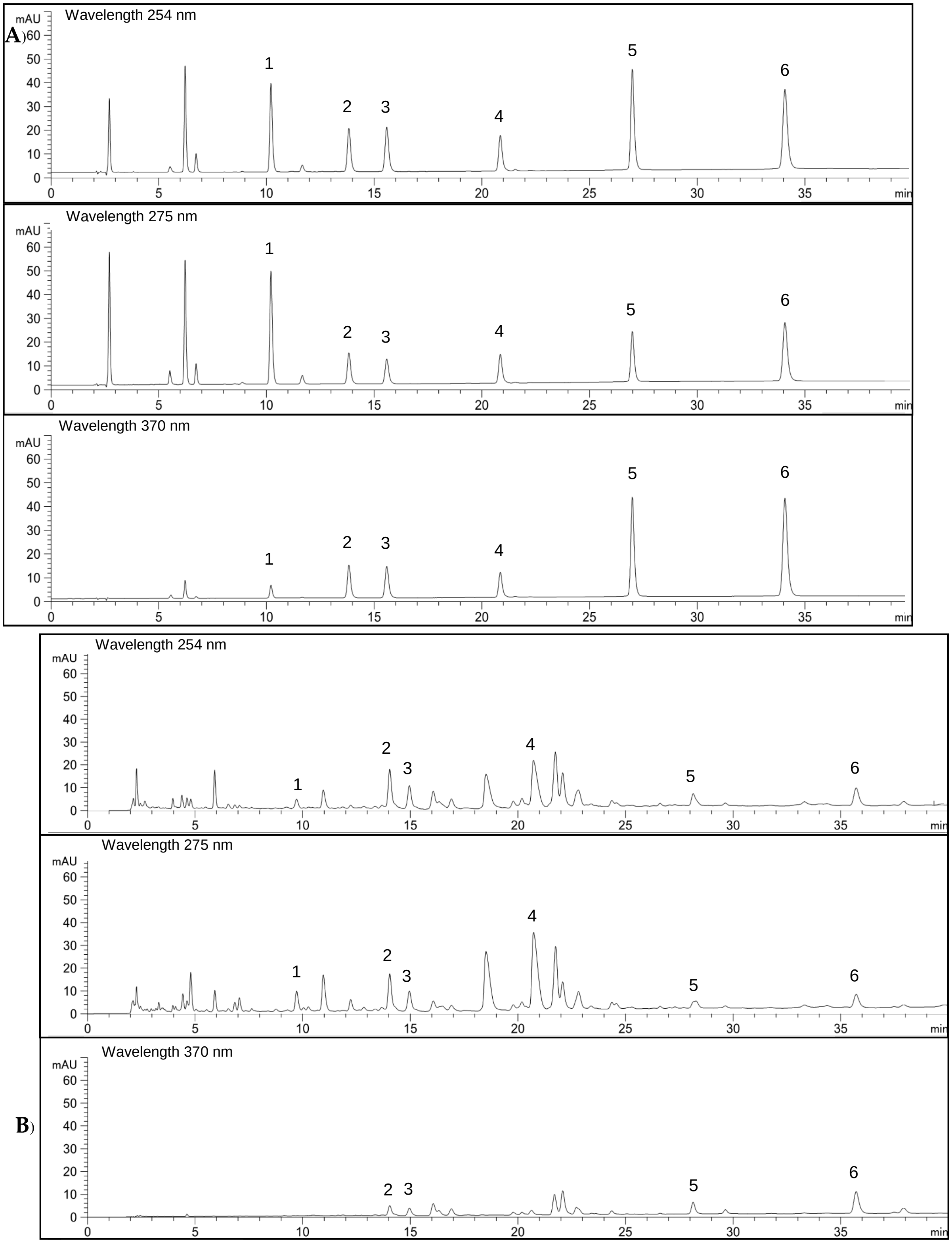

A)
Wavelength 254 nm
Wavelength 275 nm
Wavelength 370 nm
B)
Wavelength 254 nm
Wavelength 275 nm
Wavelength 370 nm
mAU
min
